# Reasons for Eating Animal Food

**Published:** 1812-01

**Authors:** 


*To the Editors of the Medical and Physical Journal.*

GENTLEMEN,

ON reading, in your Number for September, an article entitled "the Writer's Reasons for not eating Animal Food," I amused myself by putting to paper the following observations in answer; but, as it did not appear to me that the original diatribe contained such strength of argument or authority as to have any weight with the public, I did not think it worth while to trouble you on the subject. But, as it is publicly known that one physician enforces this doctrine by both precept and example; and, as the public has been lately amused by a book which tells us that a gentleman and lady have actually carried this system for several years into effect in their family, and as it is possible that some other children and dependants may be unfortunate enough to have a regimen imposed on them by the weakness or credulity of well-meaning superiors, which may probably debilitate their constitutions for life, I think it worth while to send to you for insertion my

*Reasons for eating animal food.*

1. Because, finding myself in this world without any act of volition or consciousness, and therefore evidently under the influence of some superior power, I feel it my duty humbly to acquiesce in the laws of nature.

2. Because observation and experience shew that many animals were designed by nature to support their own existence by the destruction of others. Nature herself therefore has demonstrated that it is justifiable to cause death for this very purpose.

3. Because I find it very agreeable to my own nature to partake of a good leg of mutton or fine sirloin of beef, whenever I am fortunate enough to come within knife-and-fork distance of them; and because I observe the same propensity in children, even before they can reason.

4. Because no argument can be drawn against eating animal food, from the circumstance that the flesh of all animals is not consonant to the human stomach, any more than than against the eating of vegetables, because flowers, grass, and trees, are not digestible by man.

5. Because nature has provided, and with a liberal hand offers to us, animals which seem created for this very purpose.

6. Because it is evident that every animal receives its life only on the condition that it should again give it up; and, as there seems strong reason to believe that they are merely acted on by external things and circumstances, without the power within themselves of consciousness or reflection, the mere privation of existence, excluding the manner in which it may be done, is to them no evil.

7. Because the personal experience of nearly the whole human race coincides with my own, in proving the use of animal food, wholesome, agreeable, and natural.

8. Because no argument can be fairly drawn against animal diet, from the alleged cruelty of carnivorous animals. It might be sufficient to observe that they act as well as ourselves, agreeably to the laws of nature, and therefore must act right, whether we can prove it or not. But we presume that a carnivorous animal is no more sensible that he is commiting an act of cruelty while he is devouring another, than a sheep is in eating turnips.

9. Because culinary practices are not of necessity cruel, and it is as unphilosophical as it is common and unjust to argue against any thing from its abuse.

10. Because talking of sentimental sympathy for the death of an ox, seems rather ridiculous. Nobody supposes that the brute would feel any thing like a mutual sentiment, and simple death has been proved to be no evil to a beast. The proper sentiment for man to entertain towards other animals, is to be kind and careful of them while they live. He need not concern himself about the period of their death.

11. Because nature has given to mankind ingenuity to invent various modes of dressing and preparing the flesh of animals, by which it becomes more nutritive and pleasing to the palate of people in general, than any other substances which can be employed for constant food.

12. Because man thus gives opportunity of existence and consequent enjoyment, to a much more numerous class of beings than himself. Were he to employ vegetable food only, his country would appear comparatively desolate and uninhabited, except by selfish, solitary, man.

13. Because there is almost no part of an esculent animal which is not fit for food, or employed to some useful purpose, and because it contains more nourishment in less pace than any other kind of aliment.

14. Because the killing of animals, for food, is abundantly justified by physical benefit, moral expediency, and by real necessity.

15. Because animals are endowed with such extensive powers of procreation, that, if they were not killed, man would soon not find room for himself and his vegetable plantations, on the face of the earth. The same arguments apply to the killing and eating of birds, as of other land animals. And, as to fish, Nature did not make them so delicious to the appetite of man in vain; and beyond all others they can repair the ravages committed by man on their species.

16. Because science and philosophy confirm the dictates of unbiassed nature in the appetite for this sort of food. Human and comparative anatomy, together with the soundest physiology, prove, from the structure and functions of the whole alimentary canal in man, that he was designed to employ the flesh of other animals as part of his diet. The odour is made pleasing to his olfactory nerves, his teeth were made to bite it, his palate to relish it, his stomach to digest it, and his various secretions and vital powers to assimilate it to his own nature and constitution. Nor is there the least proof of its moderate use, (and reason is given us expressly to guide our employment of every thing in this world,) being inimical to the attainment of every possible degree of excellence and virtue. Its lawfulness also might be proved by the vision of St. Paul, and many other parts of the bible, if farther proof could be deemed wanting.

I am, Gentlemen,

Your very humble servant,

